# Dimethyl fumarate is an allosteric covalent inhibitor of the p90 ribosomal S6 kinases

**DOI:** 10.1038/s41467-018-06787-w

**Published:** 2018-10-19

**Authors:** Jacob Lauwring Andersen, Borbala Gesser, Erik Daa Funder, Christine Juul Fælled Nielsen, Helle Gotfred-Rasmussen, Mads Kirchheiner Rasmussen, Rachel Toth, Kurt Vesterager Gothelf, J. Simon C. Arthur, Lars Iversen, Poul Nissen

**Affiliations:** 1 Danish Research Institute of Translational Neuroscience – DANDRITE, Nordic-EMBL Partnership for Molecular Medicine, Department of Molecular Biology and Genetics, Aarhus University, Gustav Wieds Vej 10C, DK-8000 Aarhus C, Denmark; 2 Department of Dermatology, Aarhus University Hospital, P.P. Oerumsgade 11, DK-8000 Aarhus C, Denmark; 3 Department of Chemistry and iNANO, Aarhus University, Gustav Wieds Vej 14, DK-8000 Aarhus C, Denmark; 4 Department of Clinical Medicine, Aarhus University, P.P. Oerumsgade 11, DK-8000 Aarhus C, Denmark; 5 Division of Cell Signaling and Immunology and University of Dundee, Dow Street, Dundee, DD1 5EH UK; 6 Medical Research Council Protein Phosphorylation Unit, School of Life Sciences, Wellcome Trust Building, University of Dundee, Dow Street, Dundee, DD1 5EH UK

## Abstract

Dimethyl fumarate (DMF) has been applied for decades in the treatment of psoriasis and now also multiple sclerosis. However, the mechanism of action has remained obscure and involves high dose over long time of this small, reactive compound implicating many potential targets. Based on a 1.9 Å resolution crystal structure of the C-terminal kinase domain of the mouse p90 Ribosomal S6 Kinase 2 (RSK2) inhibited by DMF we describe a central binding site in RSKs and the closely related Mitogen and Stress-activated Kinases (MSKs). DMF reacts covalently as a Michael acceptor to a conserved cysteine residue in the αF-helix of RSK/MSKs. Binding of DMF prevents the activation loop of the kinase from engaging substrate, and stabilizes an auto-inhibitory αL-helix, thus pointing to an effective, allosteric mechanism of kinase inhibition. The biochemical and cell biological characteristics of DMF inhibition of RSK/MSKs are consistent with the clinical protocols of DMF treatment.

## Abstract (web summary)

Dimethyl fumarate (DMF) is a major drug in the treatment of psoriasis and multiple sclerosis. Here the authors reveal a mechanism of RSK/MSK inhibition through covalent inhibition that can explain the observed clinical effects and the dose–response characteristics of DMF treatment.

## Introduction

**Fig. 1:**
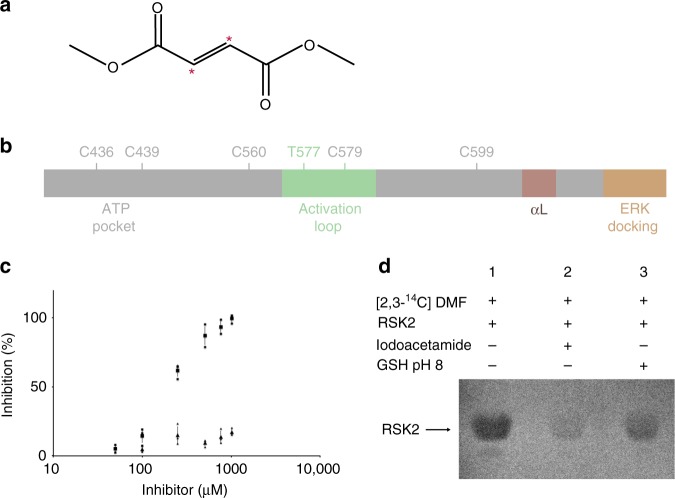
DMF inhibits RSK2. **a** DMF with Michael acceptor reactive carbons marked with red asterisks. **b** Schematic representation of RSK2^CTKD^ and position of cysteine residues. **c** Inhibition of RSK2^CTKD^ by DMF (squares) and MMF (triangles). Error bars indicate the standard deviation of mean values (*n* = 3, all data points included). **d** Covalent modification of RSK2^CTKD^ by DMF. Purified murine RSK2 (5 nmol) was incubated with ^14^C-labelled DMF (25 nmol). DMF binding is reduced by preincubation of RSK2 with 10-fold molar excess of iodoacetamide or GSH, respectively. The + and – signs represent inclusion or absence of the respective components

Systemic psoriasis treatment with fumaric acid esters has been known for >50 years, and dimethyl fumarate (DMF) is considered the clinically active agent^1^ (Fig. 1a). Most recently the European Medicines Agency (EMA) has approved a new oral formulation of DMF for the treatment of psoriasis (Procedure No. EMEA/H/C/002157/0000 and see reference^2^). DMF has furthermore proven efficacious for the treatment of relapsing-remitting multiple sclerosis (MS)^3^. The clinical response to DMF is slow, with efficacy only appearing after several weeks to months of high-dose administration^1^. Several mechanisms of action (MOA) have been proposed. DMF reacts, e.g., rapidly with glutathione (GSH)^4^ and orally administered DMF is released into the bloodstream, absorbed by the cells, and conjugated to GSH^5^. A proposed MOA for DMF treatment of MS and psoriasis therefore builds on GSH depletion increasing hemoxygenase-1 expression, impairing STAT1 (signal transducer and activator of transcription 1) phosphorylation, and hereby inhibiting Th1 and Th17 (T helper cell 1 and 17) differentiation^6,7^. However, GSH levels return to normal within 24 h of DMF administration^7^. DMF furthermore reduces neutrophil recruitment in inflammation^8^ and reduces microglial and astrocytic inflammation^9^. DMF inhibition of Th1 and Th17 differentiation has also been demonstrated through suppression of the NF-κB (nuclear factor kappa-light-chain-enhancer of activated B cells), p38 MAPK (mitogen-activated protein kinase) and ERK1/2 (extracelluar signal-regulated kinase 1 and 2) signalling pathways^8,10^. Along this line, DMF was identified as a NF-κB inhibitor^11^, and more specifically targeting the C-terminal kinase domain (CTKD) of the ribosomal S6 kinases (RSKs)^12^ and mitogen- and stress-activated kinases (MSKs)^13^.

The closely related RSKs and MSKs belong to the Ras/Raf/MAPK signalling pathway and encompass four human RSK isoforms (RSK1–4) and two MSK isoforms (MSK1 and 2)^14^. RSK1, 2, and 3 are ubiquitously expressed in human tissues and RSKs are generally recognized as regulators of proliferative processes^15^. MSK1 and 2 are expressed in most cells but at particularly high levels in cells of the immune system and the central nervous system. MSK1 and 2 display a key role in the regulation of IL-10 (interleukin 10) expression by innate immune cells^16^. RSKs and MSKs are composed of a N-terminal kinase domain (NTKD), a linker region and a C-terminal kinase domain (CTKD), and both kinase domains have adenosine triphosphate (ATP) binding sites (Fig. 1b). The RSKs are activated by phosphorylation of the activation loop in the CTKD by ERK1/2^17^, and MSKs similarly by ERK1/2 or p38 MAPK. The linker region is phosphorylated by the activated CTKD, and hereby activates the NTKD for phosphorylation of downstream targets^18^.

We find that DMF inhibits RSK/MSK kinases primarily by covalent binding to a conserved cysteine residue placed at an allosteric site, and that this interaction is likely to represent an important component of the mechanism of action of DMF as a clinical drug.

## Results

### DMF inhibition and modification of RSK2^CTKD^

The efficacy of DMF and monomethyl fumarate (MMF) as inhibitors against purified RSK2^CTKD^ and mutants hereof was evaluated in a well-established, time-resolved fluorescence resonance energy transfer (FRET) based kinase activity assay (see Methods). We found that DMF leads to full inhibition of RSK2 ^CTKD^, but at moderate efficacy with an IC_50, app._ of 225 μM, when incubated with RSK2^CTKD^ prior to ERK2 activation, whereas MMF displayed no inhibition of RSK2^CTKD^ (Fig. 1c). Considering the high-dose administration of DMF, the observed effect remains however relevant. Compared with MMF, the double bond in the α,β-unsaturated ester of DMF is an excellent (and twofold symmetric) Michael acceptor (Fig. 1a), which led us to consider if the inhibitory effect on RSK/MSK was due to covalent modifications of cysteine residues resulting in a dimethyl 2-*S*-succinate adduct^4^. Indeed, a covalent modification was identified by the reaction of [2,3-^14^C]-labelled DMF with the purified CTKD of RSK2 (RSK2^CTKD^), and the modification was diminished when cysteines were blocked or excess thiol was added (Fig. 1d). Covalent modification of purified RSK2^CTKD^ by DMF was further confirmed by mass spectrometry (Table 1). Modifications of multiple cysteines (C436, C560, C579, and C599) were observed by peptide mapping, although complete modification was observed only for C436 and C599.

**Table 1:** Degree of in vitro modification of cysteines in RSK2^CTKD^ by DMF on purified RSK2^CTKD^ (DMF1 mM) and in HEK293 cells (DMF 140 µM) estimated by mass spectrometry (*n* = 1)

Residue	Site	In vitro modification (%)	Cellular modification (%)
C436 and C439	ATP pocket	95.4	n.d.
C560	ATP pocket	88.3	0.7
C579	Activation loop	78.8	23.9
C599	Allosteric site	99.8	48.4

### Crystal structure determination and analysis of RSK^CTKD^-DMF

**Fig. 2:**
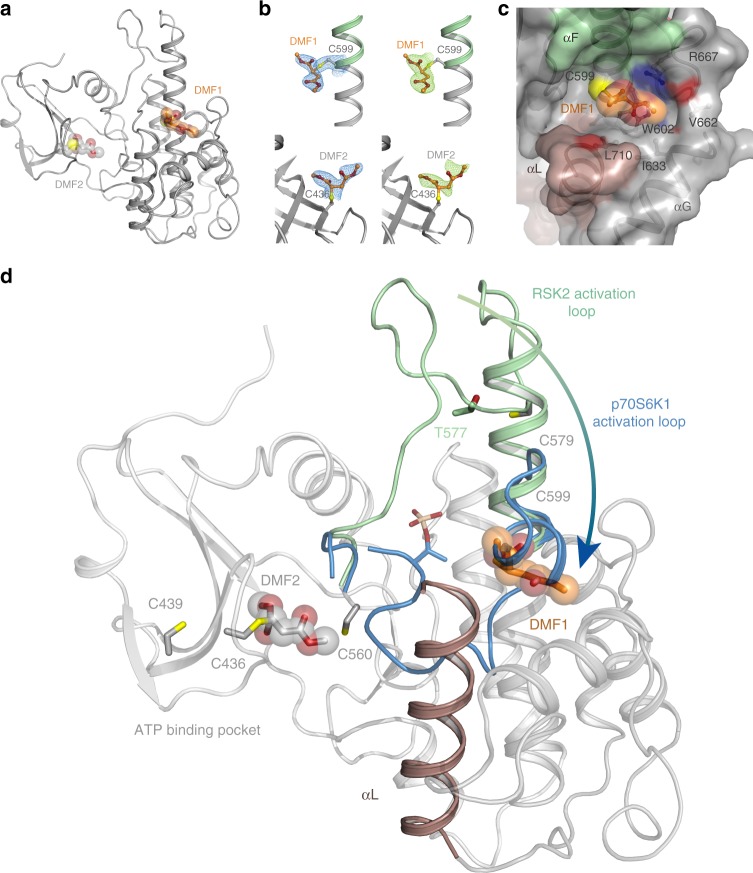
DMF binds to C436 and C599 in RSK2 crystals. **a** Cartoon representation of RSK2 with DMF covalently bound at C599 (DMF1) and C436 (DMF2). **b** Covalent binding of DMF (orange) to C599 and C436 of RSK2 (light grey). Final *2F*_*o*_*–F*_*c*_ electron density map depicted in blue represent a contour level of 1.0σ and a bias-reduced simulated annealing *F*_*o*_*–F*_*c*_ difference maps is contoured in green at 3.0 σ, both on the C436 and C599 dimethyl binding site, respectively. **c** DMF binding pocket. The regulatory αL-helix, the activation loop, nitrogens and oxygens are coloured in brown, light green, blue, and red, respectively. **d** Activation of RSK2 (light grey) leads to the phosphorylation of a threonine residue in the activation loop (light green). The movement of the activation loop has been determined by X-ray crystallography for several kinases and is shown in light blue for the related kinase p70S6K1 (PDB ID 3A62). The hinge region is undergoing large structural rearrangements during activation and covalent binding of DMF (in spheres) to C599 could abolish this by steric hindrance. A second DMF modification site, C436 was observed, but it was less well defined

We determined the structure of RSK2^CTKD^ co-crystallized with excess DMF at 1.9 Å resolution by X-ray crystallography (Table 2). Overall, the structure resembles that of the inactive RSK2^CTKD^ (PDB ID 2QR8^19^, RSMD 0.41 Å for all Cα atoms), but a DMF modification of C599 in the α-helix F (αF) was readily apparent from unbiased electron density maps and further confirmed by the refined structure, as was a less well-defined modification site at C436 at the empty ATP binding pocket (Fig. 2a, b). Focusing on the C599 site, the ester group adjacent to the two-carbon of the dimethyl 2-*S*-succinate adduct is stacked to W602, and a weak hydrogen bond is formed between the linking ester oxygen and R667 (Fig. 2c). Hydrophobic interactions are furthermore formed with I633 and L710 of αG and αL, respectively. Thus, the DMF-modified C599 side chain accommodates a rather tight binding pocket.

**Table 2:** Crystallographic data collection and refinement statistics

	Murine RSK2:DMF
Data collection^a^	
Space group	P4_1_2_1_2
Cell dimensions	
* a*, *b*, *c* (Å)	47.0, 47.0, 291.9
α, β, γ (°)	90, 90, 90
Resolution (Å)	40–1.9 (2.0–1.9)^b^
*R*_sym_ (%)	4.5 (54.9)
*R*_*p.i.m*._ (%)	1.3 (32.1)
*I*/σ*I*	23.3 (2.3)
Completeness (%)	99.4 (96.5)
Redundancy	8.6 (8.5)
Refinement	
Resolution (Å)	40–1.9
No. reflections	27,094
*R*_work_/*R*_free_ (%)	20.5/25.0
No. atoms	
Protein	2428
Ligand/ion	21
Water	121
B-factors (Å^–2^)	
Protein	64.5
Ligand/ion	80.7
Water	62.6
R.m.s. deviations	
Bond lengths (Å)	0.009
Bond angles (°)	1.44

^a^Data were collected from a single crystal

^b^Values in parentheses indicate the outer resolution bin

Following a regular activation of the CTKD by phosphorylation, the phosphorylated threonine in the activation loop forms an electrostatic interaction with a lysine of the substrate^20^, hereby exerting a pull on the activation loop. Structural comparison with the activated state of related kinases suggests that the activation loop and αF undergoes large structural rearrangements during RSK/MSK activation (Supplementary Movie 1 and Fig. 2d). The C599 site, however, is situated at a central hinge of αF, and covalent modification of C599 by DMF impairs the movement of the activation loop by steric hindrance (Fig. 2d). Displacement of the auto-inhibitory αL-helix by disruption of the hydrogen bond between S603 (αF) and Y707 (αL) are furthermore required for activation of the RSK2^CTKD^^19,20^. A stabilized interaction between αL and the C-terminal helix bundle via the hydrophobic interactions between the modified C599 and L710 of αL furthermore contribute to the DMF-bound state. However, the partially occupied DMF modification of C436 in the ATP pocket could also potentially inhibit RSK2 by blocking ATP binding (Fig. 2a, b). On the other hand, access to C579 in the activation loop was blocked by a crystal contact and therefore remained unaccounted for from the crystal structure analysis.

### In vitro studies of cysteine mutants

**Fig. 3:**
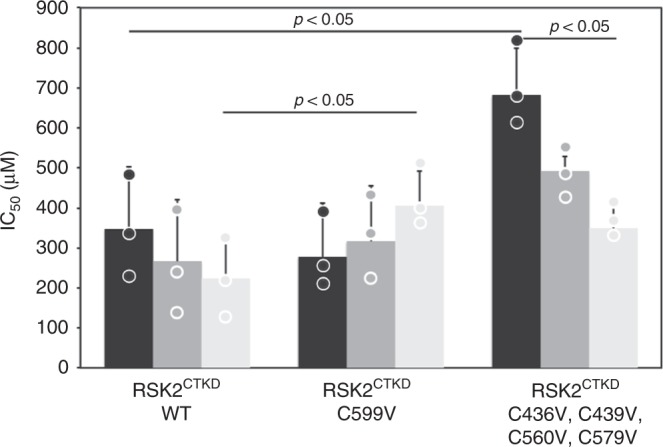
Time course of DMF inhibition. In vitro determination of apparent IC_50_ values of DMF on RSK2^CTKD^ activity following incubation with DMF at different time points (1 h: black, 24 h: dark grey, and 48 h light grey) prior to ERK2 activation (*n* = 3, all data points indicated by circles)

To further clarify the mechanism and which cysteine residues that are sufficient and responsible for the inhibitory effect of DMF, we turned to further activity assays combined with cysteine mutant forms. Cysteine mutants were designed on the basis of sequence alignments and activity assays (Supplementary Fig. 1), generally favouring cysteine-to-valine mutations over, e.g., serine and alanine substitutions. DMF displayed no efficacy against the activated form of RSK2 as to be expected from a mutual antagonism of C599 modification and activation loop docking (see above, Fig. 3). Similarly, no effect of DMF, even at 10 mM DMF saturating conditions, was observed when screened against a panel of kinases, including full-length activated RSK1, RSK2, and MSK1 (Supplementary Fig. 2). Combined mutations of all cysteines except C599 (RSK2^CTKD^ C436V, C439V, C560V, and C579V, denoted tetra-cysteine mutant) showed DMF efficacy comparable to WT over time, confirming that the allosteric site at C599 is slowly modified and resulting in a stable inhibition (Fig. 3). Mutation of C599 only (C599V) displayed the same degree of inhibition as WT following 1 h of incubation suggesting that modification of other cysteines also can cause inhibition. However, unlike for WT and the tetra-cysteine mutant this inhibition was gradually diminished over time (Fig. 3).

### Cellular studies of cysteine mutants

**Fig. 4:**
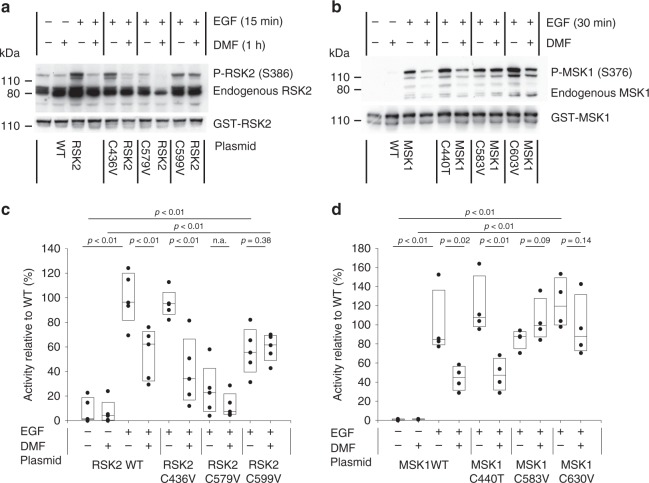
Mutational studies of DMF inhibition in cells. Mutational analysis of HEK293 cells transfected with RSK2, MSK1, or cysteine mutants hereof. HEK293 cells were supplemented with DMF (140 μM) and stimulated with epidermal growth factor (EGF, 1 ng ml^–1^). **a**, **b** Representative western blots from RSK2 and MSK1 mutational analysis respectively. **c**, **d** RSK2^CTKD^ and MSK1^CTKD^ activity determined as the autophosphorylation of S386 and S376, respectively, relative to WT. The activity of EGF-induced wild-type was set to 100% (*n* = 5 and *n* = 4, respectively; all data points indicated by black, filled circles)

We furthermore evaluated selected mutations of full-length RSK2 and MSK1 in HEK293 cells (Fig. 4). The activity of the C-terminal kinase domains was evaluated by linker region serine phosphorylation (S386 and S376 for RSK2 and MSK1, respectively) following stimulation by epidermal growth factor (EGF). DMF inhibition of RSK2 remained when C436 in the ATP binding pocket was mutated (C436V) and the same effect was observed for the C579V mutation in the activation loop although yielding lower EGF-stimulated activity. However, mutation of C599 abolished the inhibitory effect of DMF (Fig. 4a, c). Similarly, mutation of the corresponding cysteine in MSK1 (C603V) abolished the effect of DMF (Fig. 4b, d), suggesting this allosteric site is the key target for DMF in the RSKs and MSKs. Mutation of C583 in the activation loop of MSK1 also diminished the effect of DMF, which may indicate that p38 MAPK or ERK1/2 phosphorylation of the activation loop additionally is sensitive to DMF modification. Modification of this cysteine could also explain the initial DMF inhibition observed for the purified C599V mutant of RSK2^CTKD^ (Fig. 3). The cysteine in the activation loop is positioned penultimate to the threonine that is phosphorylated by upstream activating kinases, and it is solvent exposed as observed in the structure of the complex between RSK1 and ERK2^21^. The covalent modification of RSK2 at the major, allosteric site at C599 was furthermore confirmed by MS analysis and peptide mapping of HEK293 cells treated with DMF (Table 1). In cells treated with DMF, no modification is, however, observed for the C436, C439, and C560 sites, which is likely due to bound ATP present at millimolar concentration in the cell thus blocking DMF from accessing these cysteines.

## Discussion

**Fig. 5:**
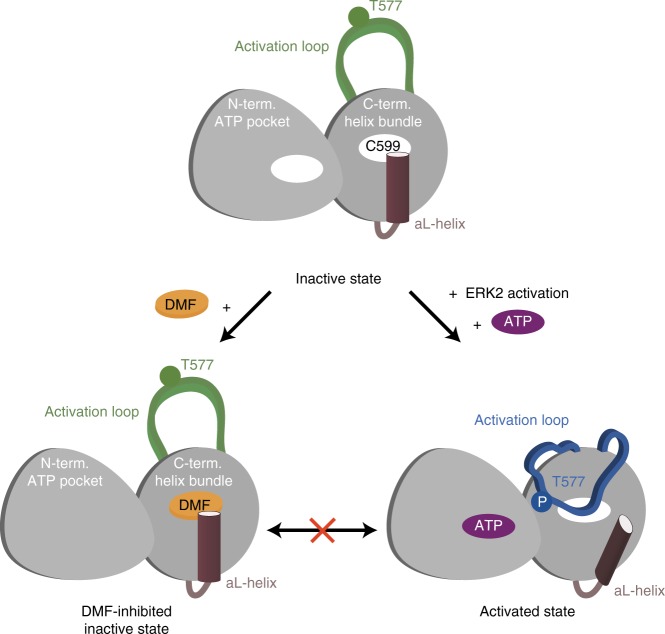
Model of DMF inhibition. Schematic figure of the activation loop transition between inactive and activated state of the C-terminal kinase domain (consisting of the ATP binding domain and the helix bundle domain). DMF targets an allosteric site and blocks kinase activation, and vice versa

The characteristics of DMF inhibition that we describe here are in good agreement with the slow response to DMF in clinical treatment^1^. We propose that the tightly packed environment of the DMF-modified C599 site (RSK2) provides a robust inhibition of RSK/MSK kinases (Fig. 5). The late onset of DMF efficacy in patients could originate from GSH competition and many other off-target reactions leading to only a slow saturation of the C599 target site. Although we have established a consistent model for DMF inhibition of RSK/MSK kinases, we also acknowledge that DMF may target additional pathways and functionalities in the cell. We note, however, that for a Michael addition to proceed, it requires a stable positioning of DMF to the target cysteine residue. Only a few kinases containing a cysteine corresponding to C599 and bearing structural resemblance to the RSK/MSK family were identified in a multiple sequence alignment of the human kinome. A closer comparison of the equivalent cysteine site in the structures or homology models of these kinases revealed that the putative DMF binding pocket diverge from that of the RSKs and MSKs and likely do provide favourable interaction sites (Supplementary Fig. 3). We therefore predict that only few specific sites entail the important, physiological effects of DMF, and that the allosteric C599 site of the RSK and MSK families is one such site. This is further augmented by the lack of inhibition observed in the broad kinase panel screen, even at 10 mM DMF (Supplementary Fig. 2).

The structural identification and characterization of the allosteric binding site at C599 invites a rational development of novel and more specific compounds based on the DMF mechanism and the efficacy of a slow off-rate due to a covalent mode of action, which from clinical applications is known to be tolerated. The reactive and sensitive nature of the allosteric site described here may also hint at a role in endogenous mechanisms of regulation of the MAPK pathway.

## Methods

### RSK2 purification

The C-terminal kinase domain of murine RSK2 (residues 400–740) including a N-terminal polyhistidine tag (His_8_), a linker (DYDIPTT) and a Tobacco Etch Virus (TEV) protease site (Glu-Asn-Leu-Tyr-Phe-Gln-Gly) in PET-22b was purchased from Genscript (RSK2-pET-22b). Val and Ser were generally the most frequent substitutions of C436, C439, C560, C579, and C599 in a multiple sequence alignment of the CamKII kinase family (Supplementary Fig. 1) and mutants were generated with the QuikChange Lightning Kit (Agilent Technologies). The Val mutants generally displayed the best ERK2 activation and were therefore used as indicated.

BL21 (DE3) Rosetta was transformed with RSK2-pET-22b and plated on lysogeny broth (LB) agar plates supplemented with 50 μg ml^–1^ ampicillin (Amp) and 35 μg ml^–1^ Chloramphenicol (Cam). Five colonies were used for inoculation of a 20 ml LB medium overnight culture supplemented with 100 μg ml^–1^ Amp and 35 μg ml^–1^ Cam. Two litres of LB, supplemented with 100 μg ml^–1^ Amp and 35 μg m^–1^l Cam, was inoculated with the overnight culture and grown at 37 °C. Expression was induced with 0.1 mM isopropyl β-d-1-thiogalactopyranoside at an optical density of A_600 nm_ = 0.8 and the temperature was lowered to 20 °C for 3 h and further lowered to 12 °C for 20 h. In all, 8 g cells were harvested from 2 l of culture and resuspended in 100 ml of lysis buffer (50 mM Tris-HCl, 100 mM NaCl and 5 mM β-mercaptoethanol, pH 7.5). Cells were lysed by high-pressure homogenization (three times at 15,000 psi) in lysis buffer supplemented with 1 mM phenylmethanesulfonyl fluoride (PMSF) and 5 μg ml^–1^ DNase I. The lysate was cleared of cell debris and aggregates by centrifugation at 25,000 *g* for 45 min. In total, 5 ml of Ni^2+^-beads slurry (Ni-sepharose 6 Fast Flow, GE Healthcare) were washed in equilibration buffer (20 mM Tris-HCl, 100 mM NaCl, 5 mM β-mercaptoethanol, pH 7.5) and incubated with supernatant for 1 h at room temperature. The supernatant and Ni^2+^-beads were poured into a Poly-Prep column (Bio-Rad) and washed with 100 ml equilibration buffer. RSK2 was eluted in two times 5 ml elution buffer (20 mM Tris-HCl, 100 mM NaCl, 5 mM β-mercaptoethanol, 500 mM imidazole, pH 7.5). The eluate was supplemented with 1 mg of recombinant TEV and immediately dialysed against 1 litre of equilibration buffer overnight at room temperature. Digested and dialysed RSK2 was loaded on the Ni-beads in the Poly-Prep column and RSK2 was collected in the flow through. RSK2 was concentrated by ultrafiltration (Vivaspin 6, 30 kDa cutoff). Protein concentration was evaluated by spectrophotometry (Nanodrop, Thermo Fisher Scientific) assuming ε_RSK2_ = 44,350 cm^−1^ M^−1^ and M_RSK2_ = 38.4 kDa. Size exclusion chromatography (SEC) was performed on a Superdex 200 10/300 GL (GE Healtcare) column in SEC buffer (10 mM Tris-HCl, 50 mM NaCl, 5 mM β-mercaptoethanol, pH 8.0) at room temperature. Fractions containing RSK2 were concentrated by ultrafiltration to 10 mg ml^−1^ (Vivaspin 6, 30 kDa cutoff) and during concentration the buffer was exchanged to 10 mM Tris-HCl, 10 mM β-mercaptoethanol, pH 8.0. RSK2 was aliqouted, flash frozen in liquid N_2_ and stored at −80 °C. RSK2 purity was evaluated by sodium dodecyl sulfate-polyacrylamide gel electrophoresis (SDS-PAGE) using a 15% separation gel. RSK2 was dialysed into reaction buffer (10 mM Tris-HCl pH 8.0 and 5 mM Tris(2-carboxyethyl)phosphine (TCEP)) in a slide-A-lyzer (Thermo Fisher Scientific) prior to reaction with DMF for ligand assays and crystallization. The purification procedure resulted > 95% pure and stable RSK2 evaluated by PAGE in SDS buffer and mass spectrometry.

### ^14^C-labelled DMF synthesis

In a flame-dried flask under argon atmosphere, 1 mg (8.5 µmol) of [2,3-^14^C]-labelled fumaric acid (55 mCi mmol^–1^, Moravek Biochemicals), and 4 mg (34 µmol) non-labelled fumaric acid were dissolved in MeOH. Subsequently, BF_3_·OEt_2_ (21 µl, 172 µmol, 4 eq.) was added and the mixture was refluxed. After 16 h, the reaction was quenched and adjusted to pH 8 via a 5% aq. NaHCO_3_ solution. The resulting mixture was extracted with dichloromethane (6 × 3 ml) and evaporated to dryness in vacuo affording DMF in 80% yield. [2,3-^14^C]-labelled DMF was dissolved in dimethylsulfoxide (DMSO) resulting in a final concentration of 100 mM. The procedure was repeated in parallel utilizing non-labelled fumaric acid. Characterization of the non-labelled DMF was performed. The obtained NMR data were in accordance with known literature values for DMF. ^1^H NMR (400 MHz, CDCl_3_) δ 6.85 (s, 2H), 3.80 (s, 6H); ^13^C NMR (100 MHz, CDCl_3_) δ 165.5, 133.5, 52.4.

### Radiolabelled ligand assays

Reactions were performed in reaction buffer in a total volume of 25 μl. In all, 5 nmol of RSK2 dialysed into reaction buffer was mixed with 25 nmol [2,3-^14^C]-labelled DMF and incubated for 30 min. Control experiments were performed by preincubation of 5 nmol RSK2 with 50 nmol iodoacetamide (Sigma-Aldrich) and 25 nmol [2,3-^14^C]-labelled DMF with 50 nmol reduced GSH pH 8.0 (Sigma-Aldrich) for 30 min prior to reaction with [2,3-^14^C]-labelled DMF and RSK2 respectively. Covalent modification of RSK2 by [2,3-^14^C]-labelled DMF was evaluated by PAGE-SDS using a 15% separation gel. A storage phosphor screen (Molecular Dynamics) was exposed to the gel for 48 h and scanned in a Typhoon Trio (GE Healthcare). The experiment was repeated three times (*n* = 3) and a representative blot is depicted.

### Crystallization

The RSK2–DMF complex was formed by incubating RSK2 (8 mg ml^–1^) dialysed into reaction buffer with 5 mM DMF for 30 min at room temperature. Aggregates were removed by centrifugation at 15,000 *g* for 5 min. Initial screening was performed using Index screen (Hampton Research) where 1 μl RSK2 was mixed with 1 μl reservoir solution and equilibrated against 500 μl reservoir using the sitting-drop vapour-diffusion method at 19 °C. An initial crystal hit was obtained in condition #43 (0.1 M Bis-Tris pH 6.5 and 25% (w/v) polyethylene glycol (PEG) 3350). The size and diffraction properties of the crystals were optimized with Additive Screen HT (Hampton Research) with condition #19 (0.05 M NaF). Crystals were reproducibly obtained in the described condition at a size suitable for data collection.

### Data collection, processing, and refinement

Crystals were mounted in nylon loops from mother liquor supplemented with 20% (v/v) ethylene glycol and flash cooled in liquid N_2_. A complete data set was collected at 100 K on the X06SA beamline at the Swiss Light Source (Paul Scherrer Institute). The diffraction images were indexed using XDS^22^ and the reflections were scaled in SCALA^23^. Molecular replacement was performed with the program PHASER^24^ and a search model derived from PDB ID 2QR8^19^. Rigid body refinement, full model refinement, and calculation of omit maps were performed in the PHENIX suite^25^. Model building and analysis was performed with Coot^26^. Root mean square deviations were calculated, superpositions were performed, and structural figures were prepared using PyMOL (Schrödinger; http://www.pymol.org). The final structure had 97% of amino-acid residues in favourable region of the Ramachandran plot, and remaining 3% in the additionally allowed region as indicated by Molprobity^27^.

### Bioinformatics

A multiple sequence alignment of kinases with at least 25% sequence identity to RSK2 was prepared with PSI-BLAST with the CTKD^RSK2^ as query against the human kinome and searched for kinases with a cysteine corresponding to C599 (Supplementary Fig. 1). A homology models of the activated state of RSK2 was prepared in Modeller^28^ based on PDB ID 3A62. The morph between the inactive state of RSK2 (PDB ID 2QR8) and the homology model of the activated state was prepared in morphinator (http://morphinator.au.dk)^29^.

### In vitro assay

In vitro activity of murine RSK2^CTKD^ was evaluated in the HTRF-KinEASE^TM^ assay (Cisbio Bioassays). Evaluation of the effect of DMF and MMF on activated RSK2^CTKD^ was performed by activation of RSK2^CTKD^ (2 μM) in reaction buffer with ERK2 (0.1 μM) (Signalchem) with 200 μM ATP and 10 mM MgCl_2_ in kinase buffer (50 mM HEPES pH 7.0, 2 mM TCEP, 3 mM NaN_3_, 0.01% BSA, and 0.1 mM Na_3_VO_4_) for 1 h at 37 °C. In all, 50 ng activated RSK2^CTKD^ was mixed with DMSO or a concentration series of DMF in kinase buffer for 1 h at room temperature in 384-well plates for fluorescence (Greiner). The reactions were started by addition of ATP (100 μM), STK1 substrate (0.1 μM) resulting in 10 μl reactions. The reactions were stopped after 20 min at room temperature with Streptavidin-XL665 in EDTA (5 μl) and STK antibody cryptate (5 μl). Evaluation of the effect of DMF on the inactive state of RSK2^CTKD^ was evaluated by incubation of RSK2^CTKD^ in reaction buffer with DMSO or a concentration series of DMF in kinase buffer followed by ERK2 activation and kinase reaction as described above. IC_50_ values were calculated by non-linear regression using sigmoid concentration response in Graph pad Prism 7. All experiments were repeated at least three times (*n* = 3) and variance and two-tailed Student's *t*-test were applied in the statistical analysis.

### Kinase panel screening

Kinase selectivity profiling for DMF was carried out as adviced (http://www.kinase-screen.mrc.ac.uk/). Briefly, protein kinase assays were carried out at room temperature (21 °C) and were linear with respect to time and enzyme concentrations under the conditions used. Assays were performed for 40 min using a Biomek 2000 Laboratory Automation Workstation in a 96-well format (Beckman Instruments, Palo Alto, CA, U.S.A.). The concentration of magnesium acetate in the assays was 10 mM, while the concentration of [γ-^33^P]-ATP (800 cpm pmol^–1^) used was selected to be close to the kinase’s *K*_*m*_ for ATP. Assays were initiated with Mg^2+^-ATP and stopped by the addition of 5 µl of 0.5 M orthophosphoric acid. Aliquots were then spotted on to P30 filtermats, washed four times in 75 mM phosphoric acid to remove ATP, once in methanol, then dried and counted for radioactivity^30^.

### Analysis of mutants

Mutational analysis of MSK1 and RSK2 was performed in a mammalian expression vector (pEBG2T) in which a glutathione *S*-transferase (GST) domain and FLAG-tag (Asp-Tyr-Lys-Asp-Asp-Asp-Asp-Lys) was fused to the N-terminus of human MSK1 (GST-FLAG-MSK1). The positions corresponding to C436, C579, and C599 in human RSK2 of human MSK1 (C440, C583, and C603) were mutated to Val, Ser, and Thr using the QuikChange Lightning Kit (Agilent Technologies). The Val mutants generally displayed the best ERK2 activation.

Human embryonic kidney cells (HEK293) were cultured in tissue culture flasks (150 cm^2^) to 60% confluence in Dulbecco's modified Eagle's medium (DMEM, Gibco) supplemented with 50 units ml^–1^ penicilin G (Gibco), 50 μg ml^–1^ streptomycin (Invitrogen), 5 μg ml^–1^ gentamycin (Gibco), 10% (v/v) foetal bovine serum (FBS, Gibco), and 2.5% HEPES (Gibco). Cells were trypsinated and seeded in 10 cm Petri dishes at a density of 6.5 × 10^6^ cells per dish in 10 ml DMEM supplemented with 50 μg ml^–1^ bovine pituitary growth hormone (BGH, Gibco), antibiotics (penicilin G, streptomycin, gentamycin), 2% FBS, and 2.5% HEPES and were incubated for 2 days. The culture medium was changed to DMEM supplemented with 2.5% HEPES for 16 h. Transfection with plasmids was performed as previously described^31^ with modifications. HEK293 cells were transfected using 3.5 μg plasmid DNA/dish dissolved in 250 μl Optimem (Invitrogen) and 30 μl Lipofectamine 2000 (Invitrogen) dissolved in 250 μl Optimem added together for 20 min before transferring to cells. The Lipofectamine and DNA complexes were incubated with cells for 6 h at 37 °C and 5% CO_2_. Cell culture medium was then changed back to DMEM special growth medium with BGH, antibiotics, 2% foetal calf serum, and 2.5% HEPES for 48 h.

DMF (Sigma-Aldrich) was dissolved in 40% (v/v) DMSO (Merck) resulting in a 70 mM stock solution and diluted 10-fold in culture medium. All stock solutions were freshly made 10 min before use. HEK293 cells (ATCC^®^ CRL-1573) were either left untreated or were pre-incubated with 140 μM of DMF for 1 h and stimulated^12^ for either 15 min (RSK2) or 30 min (MSK1) with 1 ng ml^–1^ of human recombinant EGF (Pepro Tech, UK). Cells were stopped after one wash with ice-cold phosphate-buffered saline and flash frozen in liquid nitrogen. Whole-cell extracts were prepared by adding 400 μl of 1 × cell lysis buffer (Cell Signaling Technology) to each 10 cm dish. The 1 × lysis buffer was supplemented with 22 μl protease inhibitor cocktail (EDTA-free complete, Roche Diagnostics), and 10 µl PMSF per ml buffer. The collected samples were added 1 µl Benzonase per 400 µl buffer (MERCK, Denmark), sonicated and centrifuged for 10 min at 4 °C at 10,000 *g* and the supernatants were saved for protein determination. Equal loads of protein (50 µg) were separated on pre-cast gels, SDS-Page 8–16% (Invitrogen). Proteins were blotted onto Hybond nitrocellulose membrane (Amersham). Antibodies for western blotting were all from Cell Signaling, Beverly, MA, USA: anti-phospho-MSK1 (Ser376, #9591, 1:750), anti-phospho-RSK2 (Ser386, #9341, 1:750), and mouse anti-GST (26H1, #2624, 1:2000); HRP anti-rabbit (#7074, 1:2000), anti-mouse (#7076, 1:2000). GST-RSK2 and GST-MSK1 full-length protein of about 116 kDa were identified. Antibody binding was visualized^13^ by horseradish peroxidase-conjugated second antibody in a standard ECL™ (RPN 2106) reagent on Hyperfilm™ enhanced chemoluminescence (RPN 3103K, Amersham Bioscience), and densitometric analysis of the band intensity was carried out using Kodak one-dimensional imaging analysis software. Graphs were made with Sigma Plot v.11. STATA v.14 was used to test for normal distribution and for performing Student’s *t*-tests. A probability of *P* < 0.05 was regarded as statistically significant. Uncropped western blots are shown in Supplementary Fig. 4.

### Mass spectrometry

Purified murine RSK2 was treated with 1 mM DMF or DMSO (vehicle) and left for 2 weeks. Samples were digested with chymotrypsin. From a single peptide mapping experiment (Alphalyse A/S, Odense, Denmark) it was possible to identify C436, C560, C579, and C599 with DMF modifications, whereas no peptides containing C439 were detected.

GST-RSK2 was expressed in HEK293 cells as for the mutational analyses (see below) with the difference that HEK293 cells were transfected with 6 µg of human GST-RSK2 plasmid DNA per dish. For affinity purification, an amount of 5.6 mg total protein from not stimulated (empty vehicle) or from DMF-stimulated cells was suspended in 8 ml of 1 × Lysis buffer and was added 1 ml of glutathione-sepharose 4B beads (GE 17-0756-05, GE Healthcare Life Science) each and rotated for 30 min on ice. Glutathione-sepharose 4B beads were previously equilibrated in wash and binding buffer (100 mM Tris-Base pH 7.4, 0.15 M NaCl, 1 mM EDTA). Unbound sample was removed by centrifugation 500 *g* for 2 min. Sepharose 4B beads were washed with 10 ml of wash buffer two times and centrifuged at 500 *g* for 2 min. Proteins were eluted in two steps, step one: elution buffer of 50 mM Tris-HCl and 20 mM reduced GSH (Sigma-Aldrich), pH 8.0, and step two: elution buffer with 50 mM reduced GSH, 6 ml of each. The eluted protein samples were immediately dialysed for wash buffer and overnight in Slide-A-Lyzer 10 K Dialysis Casettes (No.66810 Thermo Fisher Scientific). The next day, proteins were freeze dried in portions of 1 ml and re-dissolved in 40 µl of 50 mM TCEP in SDS loading buffer + 60 µl of native buffer (Bio-Rad #161-0738) and analysed on PAGE-SDS 8–16% Tris-glycin gels. The proteins were stained by Page Blue Protein Staining Solution (Thermo Fisher Scientific Inc). Purity was controlled by western blotting. Proteins run on the same gel, treated by vehicle or DMF, were blotted to a nitrocellulose membrane and tested with monoclonal antibody for RSK2 (Santa Cruz Biotechnology sc-9986, 1:1000) and antibody for GST (Cell Signaling #2624S, 1:2000) to identify the full-length protein of about 116 kDa. Step two elution contained a bulk amount of GST-RSK2 and gel pieces were cut out. After in-gel digestion by trypsin or chymotrypsin and by nano-HPLC-ESI-MS/MS (Proteome Factory, Berlin), DMF modifications of the five cysteine peptides were identified by the chymotrypsin digests and comparing signals from the unmodified peptides in the vehicle with DMF-treated samples in a single comparison experiment (*n* = 1). The degree of modification of the peptide containing C436 and C439 could not be determined due to the hydrophilic nature of this peptide.

## Electronic supplementary material

Supplementary Information

Description of Supplementary Video

Supplementary Movie 1

## Data Availability

The data that support the findings of this study are available from the corresponding author upon request. The refined coordinates and crystallographic data are available in the Protein Data Bank (PDB) with access code 5O1S.
