# Supplementary material for: Dimethyl fumarate is an allosteric covalent inhibitor of the p90 ribosomal S6 kinases
Source: Nat Commun. 2018 Oct 19;9:4344. doi: 10.1038/s41467-018-06787-w (PMC6195510; doi:10.1038/s41467-018-06787-w)
Supplement: Supplementary file 1 — Supplementary Information [file 41467_2018_6787_MOESM1_ESM.pdf]

Supplementary information

Dimethyl fumarate is an allosteric covalent inhibitor of the p90 ribosomal S6 kinases.

Andersen *et al*. 2018

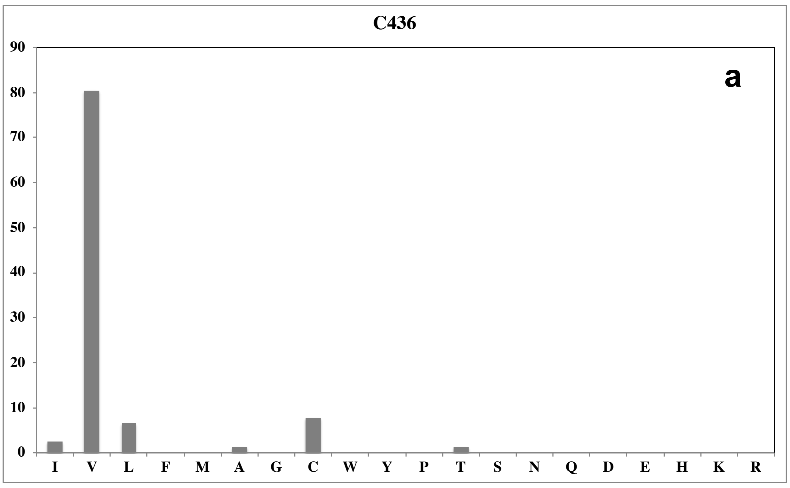
C436
a
90
80
70
60
50
40
30
20
10
0
I V L F M A G C W Y P T S N Q D E H K R

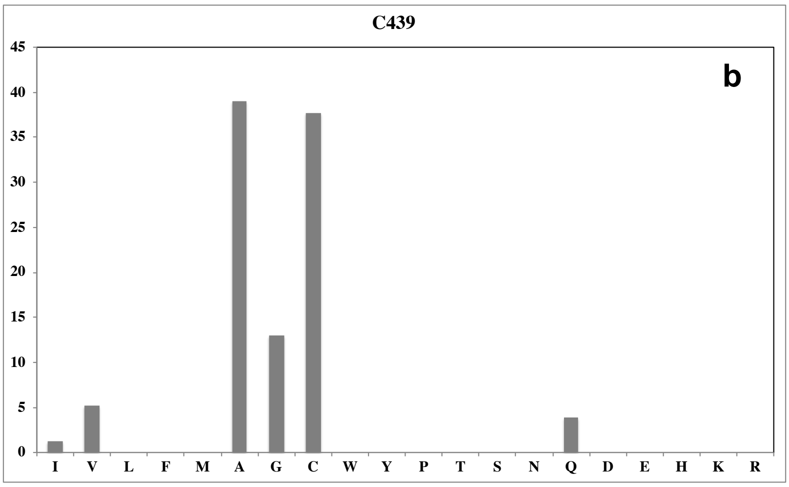
C439
b
45
40
35
30
25
20
15
10
5
0
I V L F M A G C W Y P T S N Q D E H K R

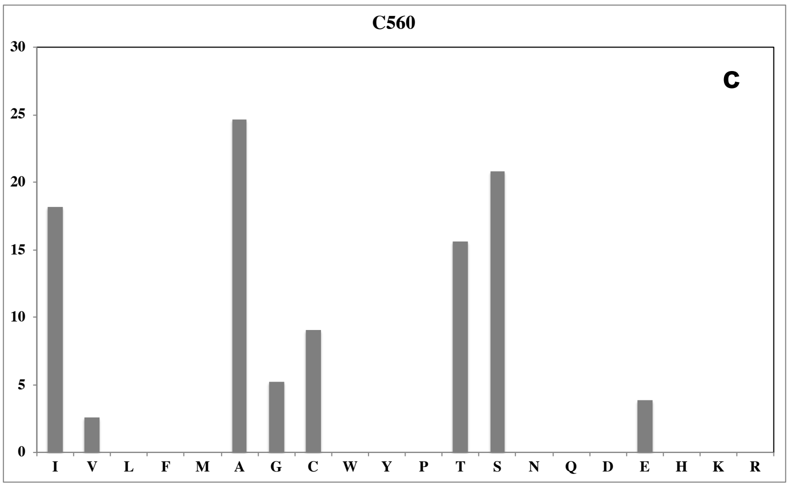
C560
c
30
25
20
15
10
5
0
I V L F M A G C W Y P T S N Q D E H K R

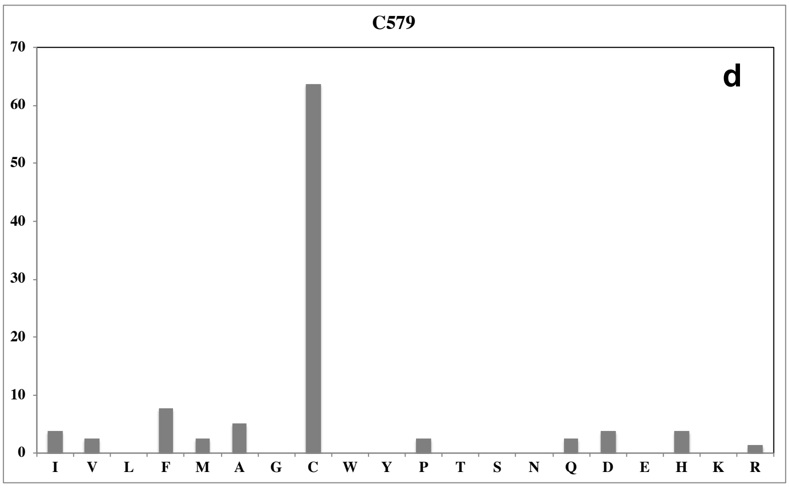

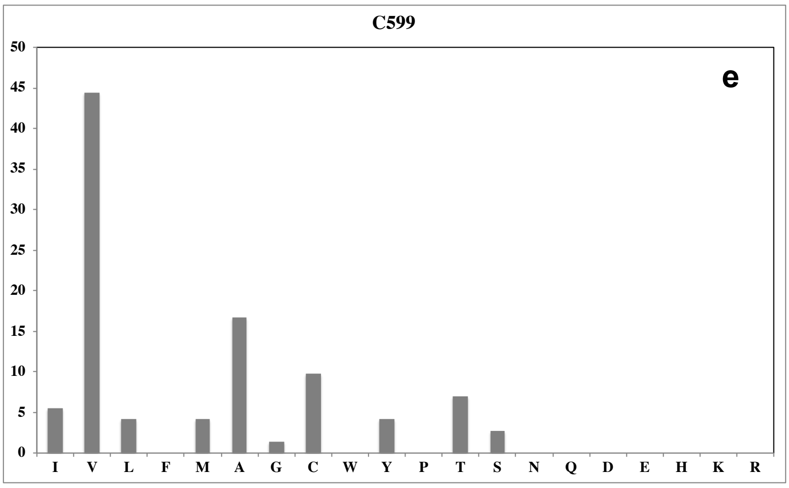

Supplementary Figure 1. Amino acid frequencies at RSK2 cysteine sites. Based on a multiple sequence alignment of the human kinases of the CamKII kinase family (58 kinases total), frequencies (Y-axis in %) for individual amino acid residues corresponding to the cysteine sites of the C-terminal kinase domain of RSK2 were derived (panels **a** through **e** for Cys436, Cys439, Cys460, Cys579 and Cys599, respectively). Accession codes used for the multiple sequence alignment are Q13131, P54646, Q8TDC3, Q8IWQ3, Q8NCB2, O14757, O96017, O14936, P53355, O43293, Q9UIK4, O15075, Q8N568Q9C098, P57058, Q14012, Q6P2M8, Q8IU85, Q9UQM7, Q13554, Q96NX5, Q13557, Q13555, Q16566, Q15139, Q9BZL6, O94806, P11801, Q96QS6, Q16644, P49137, Q8IW41, Q9P0L2, Q7KZI7, P27448, Q96L34, Q14680, Q9BUB5, Q9HBH9, Q9H1R3, Q32MK0, Q86YV6, Q8IY84, O60285, Q9H093, Q96RG2, P15735, Q16816, P11309, Q86V86, Q9P1W9, P57059, Q9Y2K2, Q9H0K1, Q9NRH2, Q9UEE5, O94768, Q15831

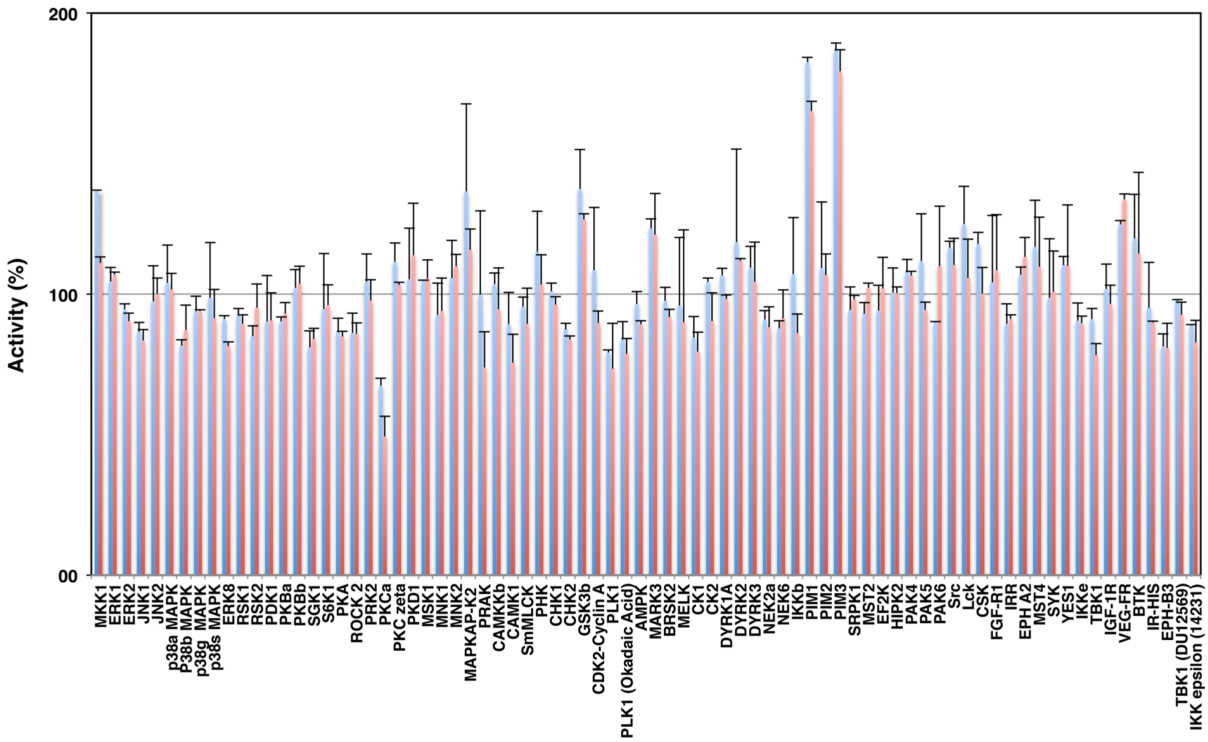

Supplementary Figure 2. **Screening of the efficacy of DMF against human kinases.** DMF was applied at 1mM (blue bars) and 10mM (red bars) against a panel of activated human kinases.

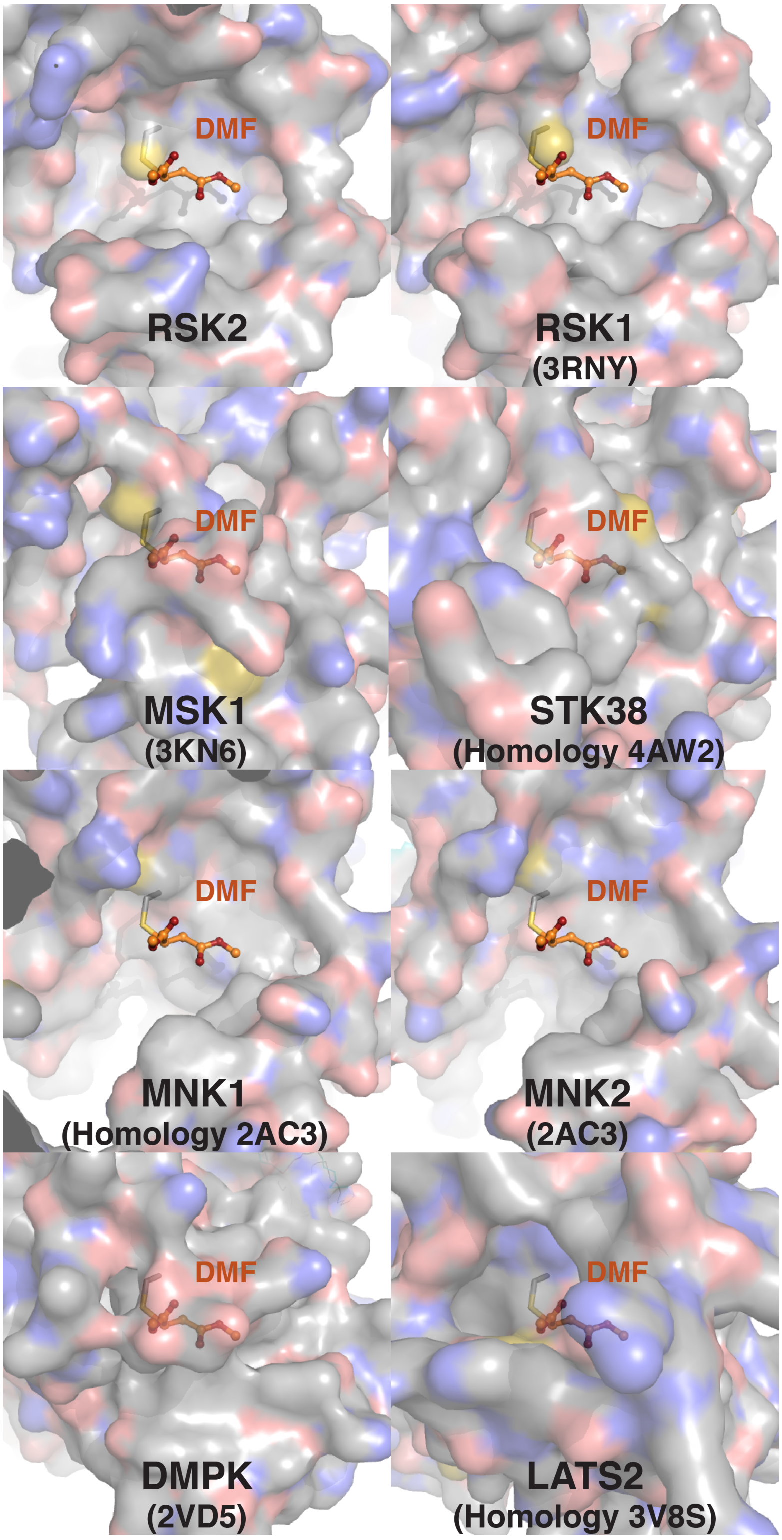

Supplementary Figure 3. **Docking of DMF at sites corresponding to Cys599 site in murine RSK2.** Comparison of a putative DMF binding site of humane kinases with a cysteine corresponding to C599 of RSK2 and sequence identity to RSK2 above 25%. The orientation of DMF when bound to RSK2 is depicted in orange sticks. MSK1 has a slightly bulkier mass over the binding site, but would still allow DMF from entering

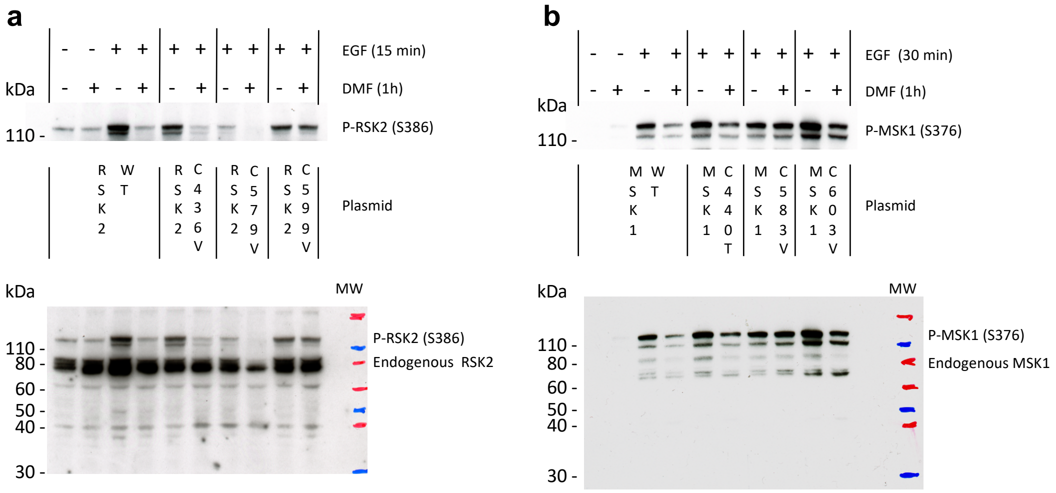

Supplementary Fig. 4. Uncropped images of western blots presented in Fig. 4.

a Representative western blot of P-RSK2 (S386) in HEK 293 cells (above), uncropped scan (below) with MW standards indicated. **b** Representative western blot of P-MSK1 (S376) in HEK 293 cells (above), uncropped scan (below) with MW standards indicated.

Supplementary References:

1. Malakhova, M. et al. Structural basis for activation of the autoinhibitory C-terminal kinase domain of p90 RSK2. *Nature Structural & Molecular Biology* **15**, 112-3 (2008).
2. Sunami, T. et al. Structural basis of human p70 ribosomal S6 kinase-1 regulation by activation loop phosphorylation. *The Journal of Biological Chemistry* **285**, 4587-94 (2010).
3. Cook, A. et al. Structural studies on phospho-CDK2/cyclin A bound to nitrate, a transition state analogue: implications for the protein kinase mechanism. *Biochemistry* **41**, 7301-11 (2002).
