# Supplementary material for: Dimethyl fumarate is an allosteric covalent inhibitor of the p90 ribosomal S6 kinases
Source: Nat Commun. 2018 Oct 19;9:4344. doi: 10.1038/s41467-018-06787-w (PMC6195510; doi:10.1038/s41467-018-06787-w)
Supplement: Supplementary file 2 — Description of Supplementary Video [file 41467_2018_6787_MOESM2_ESM.pdf]

Description of Additional Supplementary Files

File Name: Supplementary Movie 1

Description: Morph simulation between the structure of the inactive state of RSK2 (PDB ID 2QR8) 1 and a homology model of the activated state, based on the structure of the activated state of the related kinase p70S6K1 (PDB ID 3A62) 2 . The animation shows the movement of the activation loop (green) and the displacement of the auto-inhibitory αLhelix (brown) upon activation. C599 is depicted as spheres in yellow. Location of peptide substrate (blue) and ATP is superimposed from cyclin dependent kinase 2 (PDB ID 1GY3) 3 . ATP is colored by atoms with nitrogen, oxygen, carbon and phosphor in blue, red, gray and orange respectively.
